# Postoperative Nausea and Vomiting Prediction: Machine Learning Insights from a Comprehensive Analysis of Perioperative Data

**DOI:** 10.3390/bioengineering10101152

**Published:** 2023-10-01

**Authors:** Jong-Ho Kim, Bo-Reum Cheon, Min-Guan Kim, Sung-Mi Hwang, So-Young Lim, Jae-Jun Lee, Young-Suk Kwon

**Affiliations:** 1 Department of Anesthesiology and Pain Medicine, Chuncheon Sacred Heart Hospital, Hallym University College of Medicine, Chuncheon-si 24253, Republic of Korea; poik99@hallym.or.kr (J.-H.K.); qhxoddl15@hallym.or.kr (B.-R.C.); soojeongsun@hallym.or.kr (M.-G.K.); h70sm@hallym.or.kr (S.-M.H.); inooim@hallym.or.kr (S.-Y.L.); iloveu59@hallym.or.kr (J.-J.L.); 2 Institute of New Frontier Research Team, Hallym University College of Medicine, Chuncheon-si 24252, Republic of Korea

**Keywords:** nausea, vomiting, surgery, anesthesia, machine learning, prediction

## Abstract

Postoperative nausea and vomiting (PONV) are common complications after surgery. This study aimed to present the utilization of machine learning for predicting PONV and provide insights based on a large amount of data. This retrospective study included data on perioperative features of patients, such as patient characteristics and perioperative factors, from two hospitals. Logistic regression algorithms, random forest, light-gradient boosting machines, and multilayer perceptrons were used as machine learning algorithms to develop the models. The dataset of this study included 106,860 adult patients, with an overall incidence rate of 14.4% for PONV. The area under the receiver operating characteristic curve (AUROC) of the models was 0.60–0.67. In the prediction models that included only the known risk and mitigating factors of PONV, the AUROC of the models was 0.54–0.69. Some features were found to be associated with patient-controlled analgesia, with opioids being the most important feature in almost all models. In conclusion, machine learning provides valuable insights into PONV prediction, the selection of significant features for prediction, and feature engineering.

## 1. Introduction

Postoperative nausea and vomiting (PONV) is a common and distressing complication experienced by patients after surgery, particularly under general anesthesia [1,2,3,4]. This can lead to discomfort, delayed recovery, and even extended hospital stays, negatively affecting the overall patient experience and increasing healthcare costs [3,5,6]. Therefore, effective management of PONV is crucial for improving patient outcomes and satisfaction during the postoperative period [7].

Traditional approaches to managing PONV involve the administration of prophylactic antiemetic medications to high-risk patients based on clinical risk factors [7,8,9]. However, these approaches are often suboptimal as they may not accurately predict individual patient risks and can result in unnecessary medication use [10]. Consequently, there is a growing interest in developing more precise and personalized predictive models for PONV, leveraging machine learning algorithms to consider patient-specific data and risk factors.

In recent years, advancements in machine learning have revolutionized various fields, including healthcare [11,12]. In particular, machine learning holds great promise in the prediction and prevention of postoperative complications [13,14], such as PONV. The ability to accurately predict which patients are at higher risk for PONV would allow clinicians to tailor preventive strategies and interventions proactively, ultimately improving patient care and recovery.

This study aimed to present a predictive model for PONV that we developed through machine learning techniques using anonymized patient information, including demographic characteristics, medical history, surgical details, and medication administration records.

## 2. Materials and Methods

### 2.1. Study Design

This study used data that had been collected from the electronic medical records of two hospitals at Hallym University. Data were collected from 1 January 2013 to 30 April 2023. This study complied with the World Medical Association Declaration of Helsinki and was approved by the Institutional Regional Ethics Committee. The requirement for informed consent was waived because the data used were from patients whose treatment ended.

### 2.2. Participants

This study included data from patients who underwent surgery and excluded those aged <18 years who underwent surgery under non-general anesthesia, were unconscious, received postoperative ventilator care, underwent reoperation or discharge within 24 h after surgery, or had missing data.

### 2.3. Postoperative Nausea and Vomiting

PONV was defined as the occurrence of nausea or vomiting within 24 h after surgery.

### 2.4. Other Features

The dataset in this study included 103 features that consisted of patient characteristics and perioperative data: Age, the female sex, body mass index, alcohol, smoking, comorbidities (congestive heart failure, cardiac arrhythmias, valvular disease, pulmonary circulation disorders, peripheral vascular disorders, hypertension uncomplicated, hypertension complicated, paralysis, other neurological disorders, chronic pulmonary disease, diabetes uncomplicated, diabetes complicated, hypothyroidism, renal failure, liver disease, peptic ulcer disease excluding bleeding, acquired immune deficiency syndrome/human immunodeficiency virus, lymphoma, metastatic cancer, solid tumor without metastasis, rheumatoid arthritis/collagen vascular diseases, coagulopathy, obesity, weight loss, fluid and electrolyte disorders, blood loss anemia, deficiency anemia, alcohol abuse, drug abuse, psychoses, depression, gastroesophageal reflux disease, migraine), preoperative data (preoperative nausea vomiting, American Society of Anesthesiologists physical status, emergency), intraoperative data (anesthesia time, operation time, administered blood and fluid, urine output, estimated blood loss, unit of packed red blood cells, fresh frozen plasma and platelet concentration, arterial cannulation line, central venous cannulation line, Foley catheter, Levin-tube, type and dose of anesthetics, N_2_O, antiemetics and type of surgery) and postoperative data (type of patient-controlled analgesia [PCA], dose of opioid in PCA, rate of PCA, opioid dose in postanesthetic care [PACU], O_2_ supplying after surgery, frequency of administered opioid after PACU, opioid dose except for transdermal opioid patch after PACU and opioid dose of transdermal opioid patch after PACU).

### 2.5. Data Preprocessing

Data were divided into continuous and categorical categories. Continuous data were standardized by removing the mean and scaling it to the unit variance [15]. This study had an imbalance in the target PONV. There were more patients without PONV than those with PONV. In classification problems, imbalanced datasets negatively affect the accuracy of class predictions [16]. To solve this problem, we applied the synthetic minority oversampling technique (SMOTE) [17]. SMOTE is a method for generating new data of a minor class using the k-NN algorithm. Subsequently, we divided the entire dataset into training and test datasets in an 8:2 ratio. We randomly assigned similar rates of PONV to the training and test sets.

### 2.6. Machine Learning

We used five algorithms to develop the PONV prediction models. The four algorithms were as follows: logistic regression, random forest, light-gradient boosting machine, multilayer perceptron, and extreme boosting machine [18,19,20,21,22]. In the random forest, we used the balanced random forest built-in packages without SMOTE. A balanced random forest randomly under-samples each bootstrap sample to balance it [23]. Prediction models were developed by applying a training dataset to each algorithm.

Hyperparameter tuning and cross-validation using RandomSearchCV were conducted to obtain the models with the best performance. RandomSearchCV is a random combination of selected hyperparameters used to train the model [24]. The hyperparameters used in RandomSearchCV are summarized in Listing A1 in Appendix A. We determined a strategy to evaluate the performance of the five-fold cross-validated model on the training set as the area under the receiver operating characteristic curve (AUROC). Subsequently, the best models for each algorithm were evaluated using a test set.

Additionally, we developed simplified models that included features known to be associated with PONV in adults, which included female sex, smoking status, age, volatile anesthetics, duration of anesthesia, postoperative opioid use, risky surgery (laparoscopic surgery and obstetric gynecologic surgery), and preventive antiemetics. Although most known risks or mitigation factors follow the Fourth Consensus Guidelines for the Management of Postoperative Nausea and Vomiting [9], some features were missing or insufficient. Postoperative opioid use was determined when opioids were used within 24 h after surgery. Preventive antiemetics were determined when antiemetics were used intraoperatively or in the PACU before the occurrence of PONV. As we did not have data associated with a history of PONV, or motion sickness, we added data regarding preoperative nausea and vomiting. For risky surgeries, we included only laparoscopic surgery and obstetric and gynecologic surgery because we did not have data on cholecystectomy and bariatric surgery.

To obtain the feature importance, we used mutual information, which quantifies the dependency or association between two random variables. In the context of feature importance, mutual information is used to measure the amount of information gained regarding a target variable by knowing the value of a particular feature. This is a method to assess the relevance of a feature in predicting a target variable [25].

### 2.7. Statistics

Descriptive analyses were performed to compare the characteristics and perioperative data of the training and test sets. Categorical features were presented as numbers and percentages, and continuous features were presented as medians and interquartile ranges. The differences were evaluated as absolute standardized differences. Five metrics were calculated to assess the model performance; the AUROC was used as the primary metric, as well as recall, precision, f1-score, and accuracy. Bootstrapping (n = 1000) was performed to calculate 95% confidence intervals (CI). Python (version 3.7; PSF, Beaverton, OR, USA) was used to calculate the model metrics.

## 3. Results

**Figure 1:**
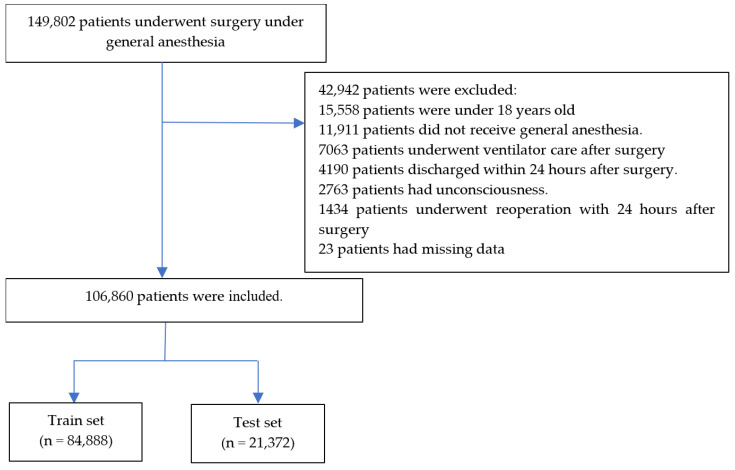
Flow chart.

A total of 149,802 patients underwent surgery under general anesthesia from 1 January 2013 to 30 April 2023. After 42,942 patients were excluded, data of 106,860 patients were divided into training (n = 84,888) and test (n = 21,372) sets. Details are summarized in Figure 1. The numbers of PONV cases were 12,287 (14.5%) and 3072 (14.4%) in the training and test sets, respectively. Patient characteristics and perioperative data are summarized in Table 1 and Table 2, respectively. The absolute standardized difference between the training and test sets was below 0.1 for all features.

### 3.1. Performance of Models, including All Features

**Figure 2:**
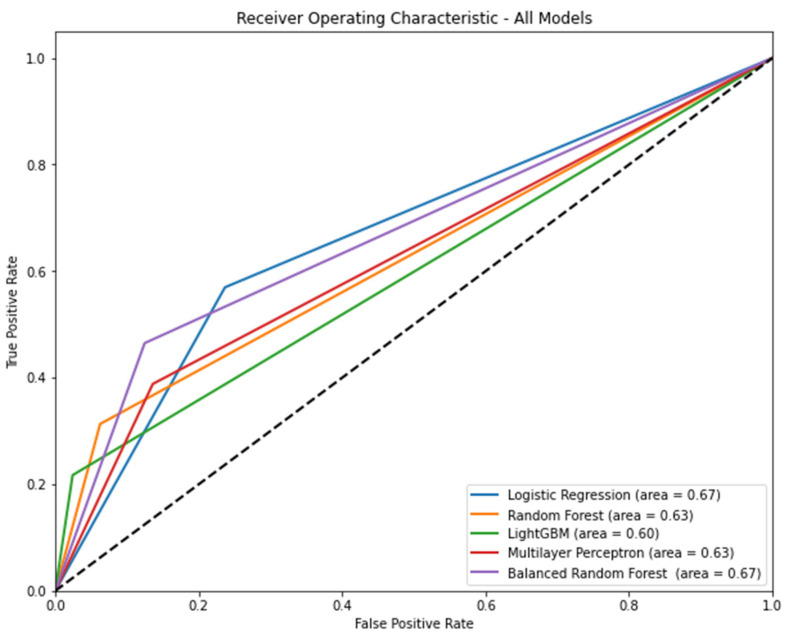
The area under the receiver operating characteristic curve of each model according to algorithm when all features are included. Note: 95% confidence interval: logistic regression, 0.66–0.68; random forest, 0.62–0.63; light gradient boosting machine, 0.59–0.60; multilayer perceptron, 0.62–0.64; balanced random forest, 0.66–0.68.

Figure 2 shows the AUROC for each model according to the algorithm. Logistic regression (AUROC [95% CI] = 0.67 [0.66–0.68]) and balanced random forest (AUROC [95% CI] = 0.67 [0.66–0.68]) had the highest AUROC. Table 3 shows the precision, recall, accuracy, and f1 score of each model according to the algorithm. In terms of precision, light GBM was the highest (0.60, 95% CI: 0.57–0.63). In terms of recall, logistic regression was the highest (0.57, 95% CI: 0.55–0.59). In terms of accuracy, light GBM was the highest (0.87, 95% CI: 0.86–0.87). In terms of f1 score, the balanced random forest was the highest (0.42, 95% CI: 0.41–0.44).

### 3.2. Performance of Models, including 10 Known Risks and Mitigating Factors

**Figure 3:**
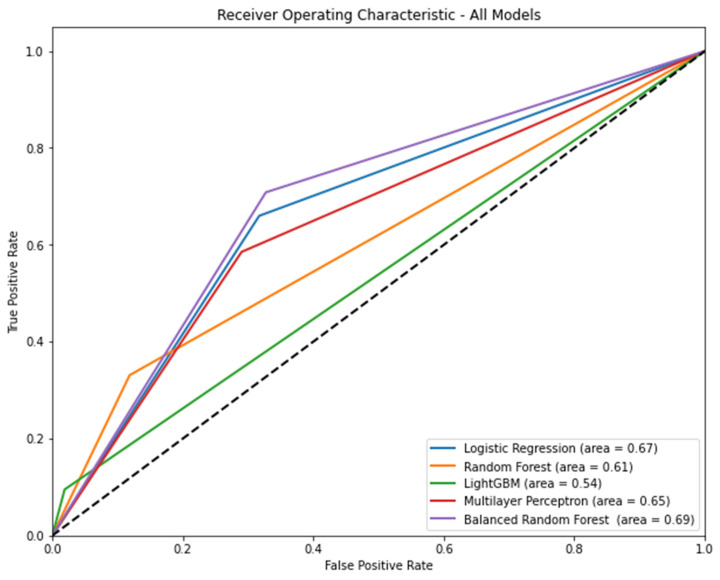
The area under the receiver operating characteristic curve of models, including known risk and mitigating factors according to the algorithm Note: 95% confidence interval: logistic regression, 0.66–0.68; random forest, 0.60–0.61; light gradient boosting machine, 0.53–0.54; multilayer perceptron, 0.64–0.66; balanced random forest, 0.68–0.70.

Figure 3 shows the AUROC of the models, including the known risks and mitigating factors according to the algorithm. Balanced random forest (AUROC [95% CI] = 0.69 [0.68–0.70]) had the highest AUROC. Table 4 shows the precision, recall, accuracy, and f1 score of each model according to the algorithm. In terms of precision, light GBM was the highest (0.46, 95% CI: 0.42–0.49). In terms of recall, balanced random forest was the highest (0.71, 95% CI: 0.69–0.72). In terms of accuracy, light GBM was the highest (0.85, 95% CI: 0.85–0.86). In terms of the f1 score, the balanced random forest was the highest (0.39, 95% CI: 0.38–0.40).

### 3.3. Feature Importance

Table 5 lists the top 20 most important features in the models. The female sex, smoking status, obstetric and gynecologic surgery, and factors associated with postoperative opioid use were included in the five models. The importance of all features is summarized in Table A1 in Appendix B.

Table 6 shows the feature importance and score in the models that include 10 known risks and mitigating features. The female sex had the highest score in the three models (logistic regression, light gradient boosting machine, and balanced random forest), whereas postoperative opioids had the highest score in the two models (random forest and multilayer perceptron).

In this study, we developed PONV prediction models with machine learning using the characteristics and perioperative data of 84,888 patients. In the evaluation of models using data from 21,372 patients, the performance of the models showed that AUROC ranged from 0.6 to 0.67 when all features were included. When the known risk and mitigating factors were included, the AUROC ranged from 0.54 to 0.69.

Shim et al. recently reported the prediction of PONV using machine learning in patients undergoing intravenous PCA [26]. Their study included 2149 patients and used seven algorithms and 13 features. Despite the small size of their data compared with ours, their AUROC ranged from 0.576 to 0.686 and was 0.643 when only Apfel risk factors were used. Their AUROC values were similar to those obtained in our study. On the other hand, Xie et al. also reported the probability of PONV for PCA using machine learning. Although they included 2222 patients and 21 features, their best AUROC value was 0.947. However, because their study included only patients who received PCA and the PCA regimen was limited, their models could not predict all patients undergoing general anesthesia. Zhou et al. reported the prediction of early postoperative PONV using multiple machine-learning and deep-learning algorithms [27]. Their study included 2149 patients and used seven algorithms and 15 features. They also had a small amount of data, but the AUROC values of the models ranged from 0.611 to 0.732. Some models showed better performance than ours. However, their data were obtained 10–15 years ago, and there were no recent data. Therefore, their models do not reflect the recent situation of anesthesia and surgery.

To develop models that can be applied to most patients under general anesthesia as much as possible, the training of the models included data from over 80,000 patients from two hospitals and 102 features. Additionally, we developed brief models that included only 10 known risks and mitigating factors. These factors are general categories that medical staff investigate or apply to general anesthesia. However, no model with excellent performance included only the 10 known risks and mitigating factors. In addition, the performance of some metrics was worse than that of models that included all features. If the removed features contain crucial information related to the target variable, their removal can result in poor performance. In this case, the model may lack the information necessary to make accurate predictions [28].

In models that included all features, the most important features were associated with opioids or PCA. In our study, if simplified models were developed with the most important features, models would have no choice but to include only the biased types of data, such as opioids and PCA, and other risk factors for PONV would have been excluded from the models. Incorporating or transforming some features may be needed to improve performance and ease of use, such as incorporating variable factors associated with postoperative opioid use. Although feature elimination sometimes helps in understanding the data, reducing computational requirements, reducing the effect of the curse of dimensionality, and improving predictor performance [29], a larger and more representative dataset can lead to better generalization [30]. The selection and transformation of features should be performed carefully, considering the specific characteristics of the data and the problem at hand.

Upon analyzing the results of feature importance, certain features consistently emerge as influential across multiple models. For instance, female sex was the variable that consistently held a substantial influence in all models, suggesting that sex might play a significant role in PONV prediction. Similarly, smoking status was another significant factor across all models, indicating its relevance in predicting the risk of PONV. Interestingly, the variables associated with opioid use demonstrated significant importance across all models, suggesting a robust association between opioid administration and the likelihood of PONV, similar to the conventional prediction of PONV. Predictions using machine learning also underscore the need for cautious opioid management strategies to mitigate the risk of PONV.

The strengths of this study include its meticulous approach to model development by utilizing a substantial dataset of over 80,000 patients and incorporating a rich set of features. This emphasis on data quantity and feature diversity provides a robust foundation for predictive modeling. In addition, the development of comprehensive models incorporating a wide range of features and simplified models based on known risk and mitigating factors acknowledges the practical need for predictive tools that can be applied to most patients undergoing general anesthesia. The integration of artificial intelligence into such medical information creates a new opportunity to design and improve new systems beyond existing systems [31].

This study also has several limitations.

1. Our models acknowledged that including only known risk and mitigating factors did not exhibit strong performance and, in some cases, showed worse metrics than the models with all features. This limitation suggests that there may be unaccounted factors contributing to PONV that are not captured solely by known risks and mitigating factors.
2. Although our study included a substantial number of patients, data were obtained from only two hospitals. This may raise questions regarding the diversity of patient populations and medical practices, potentially affecting the generalizability of the models to other healthcare settings.
3. Some studies referenced for comparison had outdated data, which might not accurately reflect the current landscape of anesthesia and surgery. This emphasizes the importance of continuously updating the models based on recent data.
4. This study highlighted the challenges of feature selection and the potential impacts of excluding informative features. However, further insight into the specific criteria and methods used for feature selection would enhance the transparency of the model development process.

## 4. Conclusions

Our study offers a valuable contribution to the realm of predictive modeling for PONV in patients undergoing general anesthesia. However, the performance of models based solely on known risks and mitigating factors highlights the complexity of PONV prediction and the need to consider additional contributing variables. Furthermore, the origin of the dataset from two hospitals warrants cautious interpretation when considering its generalizability across diverse healthcare settings. Prediction of PONV can lead to a significant reduction in PONV incidence by personalizing anesthesia and medication plans, efficiently allocating resources, improving patient experience, and strengthening recovery protocols. This can benefit patients by minimizing discomfort as well as making healthcare delivery and resource utilization more efficient. However, improved usability and performance of the model are needed to make this a reality.

## Figures and Tables

**Table 1:** Characteristics data of patients.

Features	Train Set	Test Set	ASD
Age, year	53.0 (40.0, 66.0)	54.0 (41.0, 65.0)	0.002
Female	44,455 (52.0)	11,087 (51.9)	0.0013
Body mass index	24.2 (21.9, 26.7)	24.2 (21.9, 26.7)	0.003
Alcohol	25,679 (30.0)	6429 (30.1)	0.0008
Smoking	16,324 (19.1)	4099 (19.2)	0.0008
Congestive heart failure	3388 (4.0)	841 (3.9)	0.0003
Cardiac arrhythmias	3921 (4.6)	954 (4.5)	0.0012
Valvular disease	605 (0.7)	156 (0.7)	0.0002
Pulmonary circulation disorders	624 (0.7)	170 (0.8)	0.0007
Peripheral vascular disorders	1980 (2.3)	511 (2.4)	0.0007
Hypertension uncomplicated	10,440 (12.2)	2631 (12.3)	0.001
Hypertension complicated	4764 (5.6)	1153 (5.4)	0.0018
Paralysis	392 (0.5)	96 (0.4)	0.0001
Other neurological disorders	2874 (3.4)	715 (3.3)	0.0002
Chronic pulmonary disease	8823 (10.3)	2157 (10.1)	0.0023
Diabetes uncomplicated	6058 (7.1)	1512 (7.1)	0.0001
Diabetes complicated	6293 (7.4)	1619 (7.6)	0.0021
Hypothyroidism	2039 (2.4)	492 (2.3)	0.0008
Renal failure	3986 (4.7)	1000 (4.7)	0.0002
Liver disease	4276 (5.0)	1137 (5.3)	0.0032
Peptic ulcer disease excluding bleeding	1713 (2.0)	438 (2.0)	0.0005
AIDS/HIV	12 (0.0)	8 (0.0)	0.0002
Lymphoma	387 (0.5)	102 (0.5)	0.0002
Metastatic cancer	1173 (1.4)	302 (1.4)	0.0004
Solid tumor without metastasis	16,955 (19.8)	4347 (20.3)	0.0051
Rheumatoid arthritis/collagen vascular diseases	2219 (2.6)	555 (2.6)	0
Coagulopathy	764 (0.9)	170 (0.8)	0.001
Obesity	694 (0.8)	187 (0.9)	0.0006
Weight loss	319 (0.4)	87 (0.4)	0.0003
Fluid and electrolyte disorders	2842 (3.3)	689 (3.2)	0.001
Blood loss anemia	273 (0.3)	67 (0.3)	0.0001
Deficiency anemia	3008 (3.5)	772 (3.6)	0.0009
Alcohol abuse	1885 (2.2)	459 (2.1)	0.0006
Drug abuse	1419 (1.7)	373 (1.7)	0.0009
Psychoses	632 (0.7)	163 (0.8)	0.0002
Depression	4852 (5.7)	1213 (5.7)	0
GERD	13,123 (15.4)	3231 (15.1)	0.0023
Migraine	2436 (2.8)	560 (2.6)	0.0023
Preoperative nausea and vomiting	803 (0.9)	197 (0.9)	0.0002

AIDS/HIV, acquired immunodeficiency syndrome/human immunodeficiency virus; ASD, absolute standardized difference; GERD, gastroesophageal reflux disease.

**Table 2:** Perioperative data of patients.

Features	Train Set	Test Set	ASD
ASA PS class 2	42,789 (50.4)	10,852 (50.8)	0.0072
Emergency	15,314 (17.9)	3777 (17.7)	0.0024
Anesthesia time, hour	1.8 (1.2, 2.8)	1.8 (1.2, 2.8)	0.0102
Operation time, hour	1.2 (0.7, 2.0)	1.2 (0.7, 2.0)	0.0085
Administered blood, mL	0.0 (0.0, 0.0)	0.0 (0.0, 0.0)	0.0023
Administered Fluid, mL	500.0 (300.0, 900.0)	500.0 (300.0, 900.0)	0.0085
Administered Urine, mL	0.0 (0.0, 60.0)	0.0 (0.0, 60.0)	0.0014
Estimated blood loss, mL	0.0 (0.0, 50.0)	0.0 (0.0, 50.0)	0.0097
Intraoperative PRC, unit	0.0 (0.0, 0.0)	0.0 (0.0, 0.0)	0.0034
Intraoperative FFP, unit	0.0 (0.0, 0.0)	0.0 (0.0, 0.0)	0.002
Intraoperative PC, unit	0.0 (0.0, 0.0)	0.0 (0.0, 0.0)	0.0096
A-line	29,009 (33.9)	7289 (34.1)	0.0017
C-line	7811 (9.1)	2015 (9.4)	0.0029
Foley	30,161 (35.3)	7616 (35.6)	0.0035
Nasogastric tube	1894 (2.2)	491 (2.3)	0.0008
Fasting time, hour	11.1 (8.8, 13.6)	11.1 (8.8, 13.6)	0.001
Induction drug (propofol)	80,760 (95.1)	21,372 (94.4)	0.0009
Maintenance agent (Sevoflurane)	51,110 (60.2)	12,867 (60.2)	0.0058
N_2_O	13,463 (15.7)	3410 (16.0)	0.0021
First intraoperative antiemetics	29,585 (34.6)	7406 (34.7)	0.0034
Second intraoperative antiemetics	26 (0.0)	0 (0.0)	0.0001
Type of PCA	42,635 (49.9)	10,781 (50.4)	0.0057
Total PCA dose, mg	0.0 (0.0, 100.0)	50.0 (0.0, 100.0)	0.0106
PCA flow (mg/h)	0.0 (0.0, 2.0)	1.0 (0.0, 2.0)	0.0124
Antiemetics of PCA	42,460 (49.7)	10,734 (50.2)	0.0056
Opioid dose at PACU, mg	0.0 (0.0, 0.0)	0.0 (0.0, 0.0)	0.0078
Preventive antiemetics in PACU	8164 (9.5)	2085 (9.8)	0.0026
O_2_ supply within 24 h after surgery	11,370 (13.3)	2846 (13.3)	0.0002
Frequency of postoperative opioid rescue except for TDFP	0.0 (0.0, 1.0)	0.0 (0.0, 1.0)	0.0037
Dose of postoperative opioid rescue except for TDFP, mg	0.0 (0.0, 5.0)	0.0 (0.0, 5.0)	0.0026
Postoperative TDFP within 24 h after surgery (μg/h)	0.0 (0.0, 0.0)	0.0 (0.0, 0.0)	0.0019
Intraoperative continuous infusion dose of propofol	0.0 (0.0, 0.0)	0.0 (0.0, 0.0)	0.0091
Intraoperative injection dose of propofol, mg	99.6 (79.2, 120.0)	99.6 (79.2, 120.0)	0.0012
Intraoperative dose of etomidate, mg	0.0 (0.0, 0.0)	0.0 (0.0, 0.0)	0.0009
Intraoperative dose of ketamine, mg	0.0 (0.0, 0.0)	0.0 (0.0, 0.0)	0.0071
Intraoperative dose of thiopental sodium, mg	0.0 (0.0, 0.0)	0.0 (0.0, 0.0)	0.0051
Intraoperative dose of dexmedetomidine, mg	0.0 (0.0, 0.0)	0.0 (0.0, 0.0)	0.0075
Intraoperative dose of fentanyl, μg	0.1 (0.0, 1.0)	0.1 (0.0, 1.0)	0.0052
Intraoperative dose of alfentanil, mg	0.0 (0.0, 0.2)	0.0 (0.0, 0.2)	0.0188
Intraoperative dose of sufentanil, mg	0.0 (0.0, 0.0)	0.0 (0.0, 0.0)	0.0038
Intraoperative dose of pethidine, mg	0.0 (0.0, 0.0)	0.0 (0.0, 0.0)	0.0054
Intraoperative dose of morphine, mg	0.0 (0.0, 0.0)	0.0 (0.0, 0.0)	0.0063
Intraoperative dose of neostigmine, mg	0.0 (0.0, 2.0)	0.0 (0.0, 2.0)	0.0034
Intraoperative dose of pyridostigmine, mg	0.0 (0.0, 15.0)	0.0 (0.0, 15.0)	0.0017
Intraoperative dose of sugammadex, mg	0.0 (0.0, 0.0)	0.0 (0.0, 0.0)	0.0029
Robotic surgery	2296 (2.7)	603 (2.8)	0.0014
Laparoscopic surgery	19,225 (22.5)	4729 (22.1)	0.0036
heart surgery	35 (0.0)	11 (0.1)	0.0001
Abdomen surgery	18,948 (22.2)	4612 (21.6)	0.0072
Breast surgery	3711 (4.3)	969 (4.5)	0.002
Ear surgery	2145 (2.5)	496 (2.3)	0.0019
Endocrinologic surgery	2596 (3.0)	656 (3.1)	0.0011
Eye surgery	1768 (2.1)	448 (2.1)	0.0003
Head and neck surgery	9690 (11.3)	2383 (11.2)	0.0019
Musculoskeletal surgery	23,069 (27.0)	5867 (27.5)	0.005
Neurosurgery	2341 (2.7)	608 (2.8)	0.0011
Obstetric and gynecologic surgery	8150 (9.5)	2078 (9.7)	0.0022
Spine surgery	4707 (5.5)	1196 (5.6)	0.0009
Thoracic surgery	1628 (1.9)	373 (1.7)	0.0016
Transplantation surgery	108 (0.1)	24 (0.1)	0.0002
Urogenital surgery	6416 (7.5)	1615 (7.6)	0.0009
Vascular surgery	531 (0.6)	135 (0.6)	0.0001
Skin and soft tissue surgery	3082 (3.6)	762 (3.6)	0.0007
Other surgery	3050 (3.6)	785 (3.7)	0.0011

A-line, arterial catheter; ASA PS, American Society of Anesthesiologists physical status; ASD, absolute standardized difference; C-line, central venous line; FFP, fresh frozen plasma; post anesthesia care unit; PC, platelet concentrate; PCA, patient-controlled analgesia; PRC, packed red blood cells; TDFP, transdermal fentanyl patch.

**Table 3:** Precision, recall, accuracy, and f1 score of each model according to the algorithm.

	Precision (95% CI)	Recall (95% CI)	Accuracy (95% CI)	F1 Score (95% CI)
Logistic regression	0.29 (0.28–0.30)	0.57 (0.55–0.59)	0.74 (0.73–0.74)	0.38 (0.37–0.39)
Random forest	0.46 (0.44–0.48)	0.31 (0.30–0.33)	0.85 (0.84–0.85)	0.37 (0.35–0.39)
Light gradient boosting machine	0.60 (0.57–0.63)	0.22 (0.20–0.23)	0.87 (0.86–0.87)	0.32 (0.30–0.33)
Multilayer perceptron	0.32 (0.31–0.34)	0.39 (0.37–0.41)	0.80 (0.79–0.80)	0.35 (0.34–0.37)
Balanced random forest	0.39 (0.37–0.40)	0.46 (0.45–0.48)	0.82 (0.81–0.82)	0.42 (0.41–0.44)

CI, confidence interval.

**Table 4:** Precision, recall, accuracy, and f1 score of models, including known risk and mitigating factors according to the algorithm.

	Precision (95% CI)	Recall (95% CI)	Accuracy (95% CI)	F1 Score (95% CI)
Logistic regression	0.26 (0.25–0.27)	0.66 (0.64–0.68)	0.68 (0.67–0.69)	0.38 (0.37–0.39)
Random forest	0.32 (0.30–0.34)	0.33 (0.31–0.35)	0.80 (0.80–0.81)	0.32 (0.31–0.34)
Light gradient boosting machine	0.46 (0.42–0.49)	0.10 (0.08–0.11)	0.85 (0.85–0.86)	0.16 (0.14–0.17)
Multilayer perceptron	0.25 (0.24–0.26)	0.59 (0.57–0.60)	0.69 (0.69–0.70)	0.35 (0.34–0.37)
Balanced random forest	0.27 (0.26–0.28)	0.71 (0.69–0.72)	0.68 (0.67–0.68)	0.39 (0.38–0.40)

CI, confidence interval.

**Table 5:** Top 20 importance features using mutual information according to model.

LR	RF	LGBM	MLP	BRF
**Female**	**Intraoperative dose of fentanyl**	**Intraoperative dose of fentanyl**	**Intraoperative dose of fentanyl**	**Intraoperative dose of fentanyl**
**Intraoperative dose of fentanyl**	**Total PCA dose**	**Total PCA dose**	**Total PCA dose**	**Total PCA dose**
**Total PCA dose**	**PCA flow**	**PCA flow**	**PCA flow**	**PCA flow**
**PCA flow**	**Antiemetics of PCA**	**Antiemetics of PCA**	**Type of PCA**	**Type of PCA**
**Type of PCA**	**Type of PCA**	**Type of PCA**	**Antiemetics of PCA**	**Antiemetics of PCA**
**Antiemetics of PCA**	**Female**	**Female**	**Female**	**Preventive antiemetics in PACU**
**Smoking**	**Preventive antiemetics in PACU**	**Preventive antiemetics in PACU**	**Preventive antiemetics in PACU**	**Female**
**Obstetric and gynecologic surgery**	**Smoking**	**Smoking**	**Smoking**	Urine output, mL
Alcohol	Urine output, mL	**Obstetric and gynecologic surgery**	**Obstetric and gynecologic surgery**	**Obstetric and gynecologic surgery**
Diabetes uncomplicated	Operation time	Intraoperative injection dose of propofol	Intraoperative injection dose of propofol	Head and neck surgery
Chronic pulmonary disease	Intraoperative injection dose of propofol	Operation time, hour	Alcohol	Laparoscopic surgery
Hypertension uncomplicated	Alcohol	Anesthesia time, hour	Head and neck surgery	Estimated blood loss, mL
Head and neck surgery	Anesthesia time, hour	Head and neck surgery	Intraoperative continuous infusion dose of propofol	Administered Fluid
Diabetes complicated	Administered Fluid, mL	Alcohol	Postoperative TDFP within 24 h after surgery	Anesthesia time, hour
Intraoperative injection dose of propofol	**Obstetric and gynecologic surgery**	Urine output, mL	Age	A-line
ASA PS	Estimated blood loss, mL	Administered Fluid, mL	Hypertension uncomplicated	Abdomen surgery
**Preventive antiemetics in PACU**	Head and neck surgery	Opioid dose at PACU, mg	Maintenance agent (Sevoflurane)	Operation time, hour
Renal failure	Foley	Intraoperative dose of alfentanil, mg	Urogenital surgery	Foley
GERD	Musculoskeletal surgery	Robotic surgery	Deficiency anemia	**Smoking**
N_2_O	**Dose of postoperative opioid rescue except for TDFP, mg**	**Age**	Diabetes complicated	Urogenital surgery

Bold-expressed features were included as the top 20 features in all models. ASA PS, American Society of Anesthesiologists physical status; BRF, balanced random forest; GERD, gastroesophageal reflux disease; MLP, multilayer perceptron; LGBM, light gradient boosting; LR, logistic regression; PACU, postanesthesia care unit; PCA, patient-controlled analgesia; RF, random forest; TDFP, transdermal fentanyl patch.

**Table 6:** Feature importance and score in models that include 10 known risks and mitigating features.

	LR	RF	LGBM	MLP	BRF
Female	0.322	0.05	0.109	0.125	0.3
Smoking	0.084	0.018	0.046	0.072	0.069
Age	0.162	0.013	0.018	0.04	0.085
Volatile anesthetics	0.002	0.004	0.015	0.019	0.018
Preoperative nausea and vomiting	0.009	0	0.003	0	0
Postoperative opioids	0.259	0.062	0.092	0.162	0.25
Anesthesia time	0.068	0.028	0.027	0.044	0.096
Laparoscopic surgery	0.015	0.005	0.004	0.008	0.015
Obstetric and gynecologic surgery	0.112	0.022	0.034	0.056	0.111
Preventive antiemetics	0.037	0.005	0.007	0.005	0.024

BRF, balanced random forest; LGBM, light gradient boosting machine; LR, logistic regression; MLP, multilayer perceptron; RF, random forest.

## Data Availability

Restrictions apply to the data availability. Data were obtained from the Hallym Medical Center and made available from the clinical data warehouse with permission from the Hallym Medical Center.

## Appendix A

**Listing A1.** Definition of hyperparameter search space for each algorithm.
#Define the hyperparameter search space for each algorithm. param_dist_logreg = { ‘C’: [0.001, 0.01, 0.1, 1, 10, 100], ‘penalty’: [‘l1′, ‘l2′], ‘solver’: [‘liblinear’, ‘saga’], ‘max_iter’: [100, 200, 300, 500] } param_dist_rf = { ‘n_estimators’: [100, 200, 300, 500], ‘criterion’: [‘gini’, ‘entropy’], ‘max_depth’: [None, 5, 10, 20, 30], ‘min_samples_split’: [2, 5, 10], ‘min_samples_leaf’: [1, 2, 4], ‘class_weight’: [None, ‘balanced’, ‘balanced_subsample’] } param_dist_svm = { ‘C’: [0.001, 0.01, 0.1, 1, 10, 100], ‘kernel’: [‘linear,’ ‘poly, ‘ ‘rbf,’ ‘sigmoid’], ‘gamma’: [‘scale’, ‘auto’, 0.001, 0.01, 0.1, 1, 10, 100], } param_dist_lgbm = { ‘learning_rate’: [0.01, 0.1, 0.3], ‘n_estimators’: [100, 200, 300], ‘max_depth’: [3, 5, 7, −1], ‘num_leaves’: [31, 50, 100, 200], ‘subsample’: [0.8, 0.9, 1.0], ‘colsample_bytree’: [0.8, 0.9, 1.0], ‘reg_alpha’: [0, 0.01, 0.1], ‘reg_lambda’: [0, 0.01, 0.1] } param_dist_mlp = { ‘hidden_layer_sizes’: [(50,), (100,), (50, 50), (100, 50)], ‘activation’: [‘relu’, ‘logistic’], ‘solver’: [‘adam’, ‘sgd’], ‘learning_rate’: [‘constant, ‘ ‘invscaling, ‘ ‘adaptive’], ‘alpha’: [0.0001, 0.001, 0.01], ‘batch_size’: [16, 32, 64], ‘max_iter’: [100, 200, 300] }.

## Appendix B

**Table A1:** Feature importance and score used mutual information in models that include all features.

Logistic Regression		Random Forest		Light Gradient Boosting Machine		Multi-Layer Perceptron		Balanced Random Forest	
Feature	Score	Feature	Score	Feature	Score	Feature	Score	Feature	Score
Age, year	0.014329	Age, year	0.014209	Age, year	0.010966	Age, year	0.011432	Age, year	0.035396
Female	0.150907	Female	0.133513	Female	0.09546	Female	0.057632	Female	0.109128
Body mass index	0.010811	Body mass index	0.006313	Body mass index	0	Body mass index	0.007466	Body mass index	0.004668
Alcohol	0.055069	Alcohol	0.039759	Alcohol	0.023542	Alcohol	0.019388	Alcohol	0.032532
Smoking	0.063328	Smoking	0.068043	Smoking	0.043676	Smoking	0.025659	Smoking	0.052161
Congestive heart failure	0.01305	Congestive heart failure	0.00061	Congestive heart failure	0.006554	Congestive heart failure	0.003448	Congestive heart failure	0.008212
Cardiac arrhythmias	0.013201	Cardiac arrhythmias	0	Cardiac arrhythmias	0.000727	Cardiac arrhythmias	0	Cardiac arrhythmias	0.004049
Valvular disease	0	Valvular disease	0	Valvular disease	0.000858	Valvular disease	0.004271	Valvular disease	0
Pulmonary circulation disorders	0.001711	Pulmonary circulation disorders	0	Pulmonary circulation disorders	0	Pulmonary circulation disorders	0	Pulmonary circulation disorders	0.001633
Peripheral vascular disorders	0.011444	Peripheral vascular disorders	0.002393	Peripheral vascular disorders	0.001461	Peripheral vascular disorders	0.002524	Peripheral vascular disorders	0
Hypertension uncomplicated	0.035809	Hypertension uncomplicated	0.003582	Hypertension uncomplicated	0	Hypertension uncomplicated	0.011136	Hypertension uncomplicated	0.004505
Hypertension complicated	0.022409	Hypertension complicated	0.010994	Hypertension complicated	0.004092	Hypertension complicated	0.004717	Hypertension complicated	0.004397
Paralysis	0.004824	Paralysis	0	Paralysis	0	Paralysis	0	Paralysis	0.00206
Other neurological disorders	0.007542	Other neurological disorders	0.00373	Other neurological disorders	7.43 × 10^−5^	Other neurological disorders	0	Other neurological disorders	0
Chronic pulmonary disease	0.036637	Chronic pulmonary disease	0.003064	Chronic pulmonary disease	0	Chronic pulmonary disease	0	Chronic pulmonary disease	0.001339
Diabetes uncomplicated	0.041356	Diabetes uncomplicated	0.009213	Diabetes uncomplicated	0.007142	Diabetes uncomplicated	0.001278	Diabetes uncomplicated	0.004796
Diabetes complicated	0.032078	Diabetes complicated	0.011004	Diabetes complicated	0.00391	Diabetes complicated	0.009505	Diabetes complicated	0
Hypothyroidism	0.002325	Hypothyroidism	0.005612	Hypothyroidism	0	Hypothyroidism	0.002583	Hypothyroidism	0.001331
Renal failure	0.026439	Renal failure	0.008957	Renal failure	0	Renal failure	0.0061	Renal failure	0.00426
Liver disease	0.022072	Liver disease	0.006019	Liver disease	0	Liver disease	0.002106	Liver disease	2.02 × 10^−5^
Peptic ulcer disease excluding bleeding	0.011524	Peptic ulcer disease excluding bleeding	0	Peptic ulcer disease excluding bleeding	0.000322	Peptic ulcer disease excluding bleeding	0.002732	Peptic ulcer disease excluding bleeding	0.004694
AIDS/HIV	0.004832	AIDS/HIV	0.004172	AIDS/HIV	0.004959	AIDS/HIV	0.002683	AIDS/HIV	0.00416
Lymphoma	0	Lymphoma	0.001566	Lymphoma	0	Lymphoma	0	Lymphoma	0.000138
Metastatic cancer	0.005225	Metastatic cancer	0	Metastatic cancer	0.002773	Metastatic cancer	0.000338	Metastatic cancer	0.012946
Solid tumor without metastasis	0.021974	Solid tumor without metastasis	0.01209	Solid tumor without metastasis	0.001404	Solid tumor without metastasis	0.001608	Solid tumor without metastasis	0.00237
Rheumatoid arthritis/collagen vascular diseases	0.00187	Rheumatoid arthritis/collagen vascular diseases	0	Rheumatoid arthritis/collagen vascular diseases	0	Rheumatoid arthritis/collagen vascular diseases	0	Rheumatoid arthritis/collagen vascular diseases	0.00218
Coagulopathy	0	Coagulopathy	0	Coagulopathy	0.007293	Coagulopathy	0	Coagulopathy	0
Obesity	0	Obesity	0	Obesity	0	Obesity	0	Obesity	0.000236
Weight loss	0.003957	Weight loss	0	Weight loss	0.008786	Weight loss	0.002254	Weight loss	0
Fluid and electrolyte disorders	0.00901	Fluid and electrolyte disorders	0.000449	Fluid and electrolyte disorders	0.004403	Fluid and electrolyte disorders	0.008499	Fluid and electrolyte disorders	0
Blood loss anemia	0.000666	Blood loss anemia	0	Blood loss anemia	0	Blood loss anemia	0	Blood loss anemia	0.011309
Deficiency anemia	0.016697	Deficiency anemia	0.004104	Deficiency anemia	0.00156	Deficiency anemia	0.009742	Deficiency anemia	0
Alcohol abuse	0.013662	Alcohol abuse	0.007081	Alcohol abuse	0.008237	Alcohol abuse	0.008525	Alcohol abuse	0.000989
Drug abuse	0.012531	Drug abuse	0	Drug abuse	0	Drug abuse	0.000605	Drug abuse	0.001179
Psychoses	0	Psychoses	0.00376	Psychoses	0.003083	Psychoses	0.001452	Psychoses	0
Depression	0.005406	Depression	0.005307	Depression	0.001237	Depression	0.001572	Depression	0
GERD	0.024686	GERD	0	GERD	0	GERD	0.007851	GERD	0.001501
Migraine	0.007358	Migraine	0	Migraine	0	Migraine	0.003449	Migraine	0.00197
Preoperative nausea and vomiting	0	Preoperative nausea and vomiting	0	Preoperative nausea and vomiting	0.003574	Preoperative nausea and vomiting	0.005058	Preoperative nausea and vomiting	0.002056
Anesthesia time, hour	0.008031	Anesthesia time, hour	0.038934	Anesthesia time, hour	0.030261	Anesthesia time, hour	0.000964	Anesthesia time, hour	0.072561
Operation time, hour	0.008312	Operation time, hour	0.041784	Operation time, hour	0.031438	Operation time, hour	0.006119	Operation time, hour	0.063561
ASA PS	0.029379	ASA PS	0.006369	ASA PS	0.007577	ASA PS	0.006162	ASA PS	0.026592
Emergency	0.011446	Emergency	0.014109	Emergency	0.00387	Emergency	0.002443	Emergency	0.016669
Administered blood, mL	0.007107	Administered blood, mL	0.01352	Administered blood, mL	0.001689	Administered blood, mL	0.004122	Administered blood, mL	0.018886
Administered Fluid, mL	0.00746	Administered Fluid, mL	0.038842	Administered Fluid, mL	0.017722	Administered Fluid, mL	0.003735	Administered Fluid, mL	0.081544
Administered Urine, mL	0.013733	Administered Urine, mL	0.045319	Administered Urine, mL	0.021812	Administered Urine, mL	0.002917	Administered Urine, mL	0.099572
Estimated blood loss, mL	0.020982	Estimated blood loss, mL	0.032045	Estimated blood loss, mL	0.00701	Estimated blood loss, mL	0.002474	Estimated blood loss, mL	0.081764
Intraoperative PRC, unit	0.002815	Intraoperative PRC, unit	0.008796	Intraoperative PRC, unit	0.009146	Intraoperative PRC, unit	0	Intraoperative PRC, unit	0.014102
Intraoperative FFP, unit	0	Intraoperative FFP, unit	0.001007	Intraoperative FFP, unit	0.000711	Intraoperative FFP, unit	0	Intraoperative FFP, unit	0.001156
Intraoperative PC, unit	0.005476	Intraoperative PC, unit	0	Intraoperative PC, unit	0.000244	Intraoperative PC, unit	0	Intraoperative PC, unit	0.000352
A-line	0.000496	A-line	0.019047	A-line	0.002371	A-line	0.005451	A-line	0.069996
C-line	0.005401	C-line	0.00958	C-line	0	C-line	0.003437	C-line	0.036275
Foley	0.008587	Foley	0.022761	Foley	0.002563	Foley	0.005108	Foley	0.059241
Nasogastric tube	0	Nasogastric tube	0.004042	Nasogastric tube	0.002305	Nasogastric tube	0.000118	Nasogastric tube	0
Fasting time, hour	0.005584	Fasting time, hour	0.003814	Fasting time, hour	0.00098	Fasting time, hour	0.002977	Fasting time, hour	0.012837
Induction drug	0	Induction drug	0.013387	Induction drug	0.000604	Induction drug	0.003543	Induction drug	0
Maintenance agent	0.007787	Maintenance agent	0.013547	Maintenance agent	0.008142	Maintenance agent	0.010943	Maintenance agent	0.010467
N_2_O	0.024304	N_2_O	0.011115	N_2_O	0.007055	N_2_O	0.001988	N_2_O	0.008418
First intraoperative antiemetics	0.016601	First intraoperative antiemetics	0.007223	First intraoperative antiemetics	0.00875	First intraoperative antiemetics	0.004457	First intraoperative antiemetics	0.014732
Second intraoperative antiemetics	0.004754	Second intraoperative antiemetics	0.0051	Second intraoperative antiemetics	0.002218	Second intraoperative antiemetics	0	Second intraoperative antiemetics	0.001557
Type of PCA	0.098404	Type of PCA	0.162091	Type of PCA	0.122345	Type of PCA	0.061605	Type of PCA	0.439285
Total PCA dose, mg	0.122888	Total PCA dose, mg	0.190948	Total PCA dose, mg	0.145924	Total PCA dose, mg	0.078757	Total PCA dose, mg	0.481962
PCA flow (mg/h)	0.114928	PCA flow (mg/h)	0.182959	PCA flow (mg/h)	0.145486	PCA flow (mg/h)	0.066347	PCA flow (mg/h)	0.480247
Antiemetics of PCA	0.092322	Antiemetics of PCA	0.167905	Antiemetics of PCA	0.130546	Antiemetics of PCA	0.061012	Antiemetics of PCA	0.434438
Opioid dose at PACU, mg	0	Opioid dose at PACU, mg	0.003604	Opioid dose at PACU, mg	0.011924	Opioid dose at PACU, mg	0	Opioid dose at PACU, mg	0.012209
Preventive antiemetics in PACU	0.028454	Preventive antiemetics in PACU	0.069763	Preventive antiemetics in PACU	0.091715	Preventive antiemetics in PACU	0.044332	Preventive antiemetics in PACU	0.117608
O_2_ supply within 24 h after surgery	0.011319	O_2_ supply within 24 h after surgery	0.014543	O_2_ supply within 24 h after surgery	0.000974	O_2_ supply within 24 h after surgery	0.00047	O_2_ supply within 24 h after surgery	0.041406
Frequency of postoperative opioid rescue except for TDFP	0.001584	Frequency of postoperative opioid rescue except for TDFP	0.011442	Frequency of postoperative opioid rescue except for TDFP	0.010494	Frequency of postoperative opioid rescue except for TDFP	0.008148	Frequency of postoperative opioid rescue except for TDFP	0.033191
Dose of postoperative opioid rescue except for TDFP, mg	0.006859	Dose of postoperative opioid rescue except for TDFP, mg	0.020091	Dose of postoperative opioid rescue except for TDFP, mg	0.00501	Dose of postoperative opioid rescue except for TDFP, mg	0	Dose of postoperative opioid rescue except for TDFP, mg	0.044087
Postoperative TDFP within 24 h after surgery (μg/h)	0	Postoperative TDFP within 24 h after surgery (μg/h)	0	Postoperative TDFP within 24 h after surgery (μg/h)	0.004694	Postoperative TDFP within 24 h after surgery (μg/h)	0.011601	Postoperative TDFP within 24 h after surgery (μg/h)	0.00893
Intraoperative continuous infusion dose of propofol	0.009027	Intraoperative continuous infusion dose of propofol	0.003551	Intraoperative continuous infusion dose of propofol	0.003871	Intraoperative continuous infusion dose of propofol	0.012563	Intraoperative continuous infusion dose of propofol	0.002748
Intraoperative injection dose of propofol, mg	0.031009	Intraoperative injection dose of propofol, mg	0.040009	Intraoperative injection dose of propofol, mg	0.035606	Intraoperative injection dose of propofol, mg	0.020658	Intraoperative injection dose of propofol, mg	0.042206
Intraoperative dose of etomidate, mg	0.003061	Intraoperative dose of etomidate, mg	0.012007	Intraoperative dose of etomidate, mg	0.001926	Intraoperative dose of etomidate, mg	0.001963	Intraoperative dose of etomidate, mg	0
Intraoperative dose of ketamine, mg	0	Intraoperative dose of ketamine, mg	0.012963	Intraoperative dose of ketamine, mg	0	Intraoperative dose of ketamine, mg	0.007965	Intraoperative dose of ketamine, mg	0
Intraoperative dose of thiopental sodium, mg	0	Intraoperative dose of thiopental sodium, mg	0.009748	Intraoperative dose of thiopental sodium, mg	0	Intraoperative dose of thiopental sodium, mg	0.00023	Intraoperative dose of thiopental sodium, mg	0
Intraoperative dose of dexmedetomidine, mg	0.003159	Intraoperative dose of dexmedetomidine, mg	0.001652	Intraoperative dose of dexmedetomidine, mg	0	Intraoperative dose of dexmedetomidine, mg	0	Intraoperative dose of dexmedetomidine, mg	0.010115
Intraoperative dose of fentanyl, μg	0.139895	Intraoperative dose of fentanyl, μg	0.206067	Intraoperative dose of fentanyl, μg	0.167898	Intraoperative dose of fentanyl, μg	0.080612	Intraoperative dose of fentanyl, μg	0.493458
Intraoperative dose of alfentanil, mg	0.007663	Intraoperative dose of alfentanil, mg	0.010001	Intraoperative dose of alfentanil, mg	0.011716	Intraoperative dose of alfentanil, mg	0	Intraoperative dose of alfentanil, mg	0.03767
Intraoperative dose of sufentanil, mg	0	Intraoperative dose of sufentanil, mg	0.004908	Intraoperative dose of sufentanil, mg	0.005716	Intraoperative dose of sufentanil, mg	0.00315	Intraoperative dose of sufentanil, mg	0.00679
Intraoperative dose of pethidine, mg	0.002121	Intraoperative dose of pethidine, mg	0	Intraoperative dose of pethidine, mg	0.001803	Intraoperative dose of pethidine, mg	0	Intraoperative dose of pethidine, mg	0.009948
Intraoperative dose of morphine, mg	0	Intraoperative dose of morphine, mg	0	Intraoperative dose of morphine, mg	0	Intraoperative dose of morphine, mg	0.000745	Intraoperative dose of morphine, mg	0
Intraoperative dose of neostigmine, mg	0.011668	Intraoperative dose of neostigmine, mg	0	Intraoperative dose of neostigmine, mg	0.005509	Intraoperative dose of neostigmine, mg	0.003925	Intraoperative dose of neostigmine, mg	0.006208
Intraoperative dose of pyridostigmine, mg	0.015152	Intraoperative dose of pyridostigmine, mg	0.007306	Intraoperative dose of pyridostigmine, mg	0.006987	Intraoperative dose of pyridostigmine, mg	0.007709	Intraoperative dose of pyridostigmine, mg	0.01198
Intraoperative dose of sugammadex, mg	0.004219	Intraoperative dose of sugammadex, mg	0.002466	Intraoperative dose of sugammadex, mg	0.007416	Intraoperative dose of sugammadex, mg	0.005625	Intraoperative dose of sugammadex, mg	0.006301
Robotic surgery	0.010594	Robotic surgery	0.009638	Robotic surgery	0.011253	Robotic surgery	0.002059	Robotic surgery	0.020797
Laparoscopic surgery	0.005243	Laparoscopic surgery	0.001475	Laparoscopic surgery	0.00509	Laparoscopic surgery	0.006372	Laparoscopic surgery	0.08291
heart surgery	0.001553	heart surgery	0	heart surgery	0.003475	heart surgery	0	heart surgery	0.002751
Abdomen surgery	0	Abdomen surgery	0.006568	Abdomen surgery	0.000849	Abdomen surgery	9.77 × 10^−6^	Abdomen surgery	0.064653
Breast surgery	0.015245	Breast surgery	0.008716	Breast surgery	0.00424	Breast surgery	0.003715	Breast surgery	0.026665
Ear surgery	0.000189	Ear surgery	0.003594	Ear surgery	0	Ear surgery	0	Ear surgery	0.014613
Endocrinologic surgery	0.007103	Endocrinologic surgery	0	Endocrinologic surgery	0.007086	Endocrinologic surgery	0.001702	Endocrinologic surgery	0.01862
Eye surgery	0.003236	Eye surgery	0	Eye surgery	0.002919	Eye surgery	0.001273	Eye surgery	0.003301
Head and neck surgery	0.032772	Head and neck surgery	0.028516	Head and neck surgery	0.028426	Head and neck surgery	0.018761	Head and neck surgery	0.088718
Musculoskeletal surgery	0.016588	Musculoskeletal surgery	0.022552	Musculoskeletal surgery	0.00291	Musculoskeletal surgery	0.00477	Musculoskeletal surgery	0.044453
Neurosurgery	0.004762	Neurosurgery	0.002645	Neurosurgery	0.002558	Neurosurgery	0	Neurosurgery	0.006022
Obstetric and gynecologic surgery	0.056	Obstetric and gynecologic surgery	0.038353	Obstetric and gynecologic surgery	0.037278	Obstetric and gynecologic surgery	0.025596	Obstetric and gynecologic surgery	0.094796
Spine surgery	0.001873	Spine surgery	0.008075	Spine surgery	0.00383	Spine surgery	0.001408	Spine surgery	0.001849
Thoracic surgery	0.006697	Thoracic surgery	0	Thoracic surgery	0.003808	Thoracic surgery	0	Thoracic surgery	0.004475
Transplantation surgery	0	Transplantation surgery	0	Transplantation surgery	0	Transplantation surgery	0.000324	Transplantation surgery	0.000534
Urogenital surgery	0.018391	Urogenital surgery	0.018924	Urogenital surgery	0.005083	Urogenital surgery	0.010378	Urogenital surgery	0.045068
Vascular surgery	0.010679	Vascular surgery	0.004212	Vascular surgery	0	Vascular surgery	0	Vascular surgery	0.000859
Skin and soft tissue surgery	0.010685	Skin and soft tissue surgery	0	Skin and soft tissue surgery	0.00063	Skin and soft tissue surgery	0.003378	Skin and soft tissue surgery	0.008169
Other surgery	0.00544	Other surgery	0.010513	Other surgery	0	Other surgery	0.006076	Other surgery	0
